# The effects of propolis on pro-oxidant–antioxidant balance, glycemic control, and quality of life in chronic kidney disease: a randomized, double-blind, placebo-controlled trial

**DOI:** 10.1038/s41598-023-37033-z

**Published:** 2023-06-19

**Authors:** Paniz Anvarifard, Alireza Ostadrahimi, Mohammadreza Ardalan, Maryam Anbari, Zohreh Ghoreishi

**Affiliations:** 1 Student Research Committee, School of Nutrition and Food Sciences, Tabriz University of Medical Sciences, Tabriz, Iran; 2 Nutrition Research Center, Tabriz University of Medical Sciences, Tabriz, Iran; 3 Department of Clinical Nutrition, School of Nutrition and Food Sciences, Tabriz University of Medical Sciences, Attar-Neishaburi St., Golgasht Alley, Azadi Blvd., Tabriz, Iran; 4 Kidney Research Center, Tabriz University of Medical Sciences, Tabriz, Iran

**Keywords:** Chronic kidney disease, Randomized controlled trials

## Abstract

Chronic kidney disease (CKD) is a progressive kidney damage with an increasing prevalence. Some evidence suggests that propolis as a novel antioxidant, anti-inflammatory, and immunomodulatory agent may have beneficial effects in CKD. The aim of this study was to evaluate the efficacy of propolis on some kidney function parameters, pro-oxidant–antioxidant balance (PAB), glycemic status, quality of life, and blood pressure (BP) in patients with CKD. In this study, 44 patients with CKD were randomly assigned to receive propolis capsules at a dose of 250 mg daily or placebo for three months. Of 44 randomized individuals, 35 completed the trial. At the end of the intervention (end of month three), improvement in some dimensions of health-related quality of life (HRQoL) (derived from Kidney Disease and Quality of Life Short-Form (KDQOL-${\mathrm{SF}}^{\mathrm{TM}}$, v. 1.3) questionnaire) were significantly higher in the propolis group than the placebo group, even after adjustment for baseline values, present of diabetes, and age (*P* < 0.05). Like systolic and diastolic BP, changes in serum creatinine, 24-h urine volume and protein, fasting blood sugar (FBS), hemoglobin A1C (HbA1C), insulin, homeostasis model of assessment-insulin resistance (HOMA-IR), quantitative insulin sensitivity check index (QUICKI), and PAB did not differ significantly between the two groups (*P* > 0.05). No serious adverse events were reported throughout the study. Propolis supplementation may improve the HRQoL of CKD patients. More studies are needed to validate the adjunct use of propolis for metabolic control of CKD patients.

## Introduction

Chronic kidney disease (CKD) is a non-communicable progressive disease with a wide range of morbidity and mortality characterized by functional and structural changes in the kidney that occurs more often in patients with diabetes and hypertension (HTN)^1,2^. According to the current guidelines, CKD is described as a gradual and permanent loss of kidney function, an estimated glomerular filtration rate (eGFR) below 60 mL/min per 1.73 m^2^, or presence of markers of renal impairment, including hematuria, albuminuria, or defects detected by imaging or laboratory analysis, which last for more than three months^1^. The severity of CKD varies from kidney damage with normal function to kidney failure (or end-stage renal disease/ESRD), which defines as eGFR less than 15 mL/min per 1.73 m^2^^1^. Many cases of early-stage CKD are usually asymptomatic until the advanced stages with some clinical or sub-clinical manifestation (i.e., eGFR of less than 30 mL/min per 1.73 m^2^)^1^. Chronic kidney disease poses a substantial and growing global health burden: Some forms of CKD affects about 10% of the adult population worldwide, resulting in 1.2 million deaths and 35.8 million disability-adjusted life-years (DALYs) in 2017^3^. Premature death in People with CKD is up to ten times more likely than progression to ESRD, mainly due to cardiovascular diseases (CVD)^4^. Compared with the general population, people with CKD have a considerably lower health-related quality of life (HRQoL), and it falls in proportion to the decreasing rate of eGFR^4^. Therefore, early detection and treatment of CKD are crucial for attenuating ESRD, CVD, and total mortality^4,5^. Evidences have shown that, oxidative stress (OS), HTN, and hyperglycemia are three critical parameters for CKD pathogenesis and progression^1,6^. Although non-pharmacological interventions (e.g., lifestyle and dietary modifications) along with pharmacological treatments can be used to maintain kidney function, the development of innovative approaches can be very helpful for slowing the progression of the disease, avoiding the complications, achieving greater longevity, and enhancing HRQoL, particularly in early-stage^1^.

Propolis is a natural resinous mixture produced by honeybees (mostly Apis mellifera) by mixing exudate gathered from various plant sources with salivary enzymes and wax^7^. Bees use propolis to seal the holes in their honeycombs and cover surfaces to maintain inner moisture and temperature, provide an internal sterile environment and protect the entrance against invaders^8^. Since ancient times, propolis has been used in traditional medicine due to numerous beneficial properties^8^. The chemical composition of propolis is highly variable, depending on factors such as the season and vegetation at the collection site and the bees' species^8^. Propolis samples from different world areas have been reported to contain more than 300 active constituents, including phenolic acids and the related esters, flavonoids, terpenes, aromatic aldehydes and alcohols, stilbenes, *b*-steroids, and fatty acids^7,9,10^. Propolis and its compounds are usually well-tolerated and nontoxic when used in moderation^8^. Based on previous animal studies, ingestion of approximately 1.4 mg propolis/kg/day or 70 mg propolis/day is potentially nontoxic for the organism; however, exceeding the dose of 15 g/day may cause adverse effects^8^. The median lethal dose (LD50) of propolis extract while given to mice is higher than 7.34 g/kg, which assures human therapeutic dosage safety^11^. Many studies have demonstrated the beneficial effects of propolis on some chronic diseases due to its antibacterial, antiviral, antifungal, antiprotozoal, antioxidant, anti-inflammatory, immunomodulatory, antihyperglycemic, antihypertensive, antiproliferative, and hepatoprotective properties^7^. Recently propolis was also examined in vivo and in vitro for nephroprotective effects, with promising results^12,13^. Considering CKD pathophysiology and unique possessions of propolis, this study was aimed to assess the effects of propolis supplementation on some kidney function parameters, pro-oxidant–antioxidant balance (PAB), glycemic status, quality of life, and blood pressure (BP) in patients with CKD. Given the current evidences, experimental studies and clinical trials regarding propolis effectiveness on CKD are rare, and their results are usually controversial. Therefore, further clinical trials are required to clarify propolis efficacy in this group of patients.

## Materials and methods

### Study design

The present study was a multi-centered, randomized, parallel double-blind, placebo-controlled, phase ΙΙI clinical trial to evaluate the effect of propolis on patients with CKD. This trial was conducted according to the latest version of the declaration of Helsinki and was approved by the Medical Ethics Committee of Tabriz University of Medical Sciences (approval number: IR.TBZMED.REC.1399.177). Furthermore, it was prospectively registered at the Iranian Registry of Clinical trial (registration number: IRCT20191218045798N1) on 07/06/2020 and is available at https://en.irct.ir/trial/48603. Participants were recruited from Salamat Polyclinic and Asad Abadi Academic Hospital, Tabriz, Iran, over September 2020 to October 2021. All participating patients provided written informed consent.

### Study population

Patients aged 20–80 years who had been diagnosed with CKD due to diabetes, HTN, or another underlined reasons, with an eGFR of 30–89 mL/min per 1.73 m^2^ and body mass index (BMI) of 18.5–35 kg/m^2^ were included. The patients who underwent a kidney transplant; those who were pregnant or breastfeeding mother or professional athletes; treating with steroids or other immune system suppressors; patients with malignancy, inflammatory or infectious diseases, asthma, hepatic disorders, or severe depression; allergies to plants, especially honey bee products; those who were on any forms of herbal supplements within the past three months, and those who abused alcohol, cigarettes and drugs were excluded from the study. The sample size was calculated based on the previous clinical trial using mean ± standard deviation (SD) for eGFR^14^. Considering 95% confidence interval, α = 0.05, and 80% power, 17 CKD patients for each study group were estimated using PASS software, version 15. To compensate for an expected drop-out rate of 30%, we considered 22 patients for each group.

### Randomization and blinding

Of 220 patients evaluated, 53 were considered eligible to be enrolled in the study. Finally, 44 patients were randomly assigned in a 1:1 ratio to either propolis or placebo group. The randomization sequence was generated using a table of random numbers in permuted blocks of two, with patients stratified according to age (20–60 and 60–80 years) and present of diabetes. The propolis and placebo capsules were provided in the same organoleptic characteristics and packaging for appropriate blinding. Drug containers were labeled with two different codes for propolis or placebo by the company that manufactured the capsules. A randomization coordinator, who was not involved in the study, created an allocation sequence and provided drug containers inside the consecutively numbered, opaque, sealed envelopes to ensure concealed allocation. All research contributors and the study subjects were blind to the intervention codes and the patients’ assignment.

### Intervention and follow-up

Propolis and placebo capsules were prepared by Asal Shahdineh Golha Co., Isfahan, Iran. The propolis sample was collected during the fall season from beehives located in Isfahan, Iran, verified by an expert agricultural organization. Each propolis capsule contained 125 mg poplar type propolis ethanol extract, 187.5 mg bee pollen, and 187.5 mg oat, a total amount of 36 mg phenolic compounds (expressed as gallic acid equivalent). According to Bankova recommendation for chemical standardization of poplar type propolis^15^, total flavones/flavonols, flavanones/dihydroflavonols, and total phenolics content in propolis sample were measured using the spectrophotometric assay based on the formation of aluminum chloride complex, the colorimetric method with DNP (2,4- dinitrophenylhydrazine), and the Folin–Ciocalteu procedure, respectively. These amounts were within the recommended range: total flavones and flavonols: 8.4%, total flavanones and dihydroflavonols: 4.6%, and total phenolic compounds: 28%. Patients in the intervention group received the poplar propolis capsule twice a day (overall 250 mg propolis ethanol extract per day) for three months before breakfast and dinner. Those in the placebo group received the placebo capsule (containing 125 mg wheat starch, 187.5 mg bee pollen, and 187.5 mg oat) twice daily, based on the same protocol as the propolis group. The dosage of propolis was obtained from studies that had used similar amounts without observing side effects^14,16^. The study did not change any conventional treatment of the patients. Along with supplementation, subjects in both groups received a renal diet and were asked to follow a moderate-intensity exercise like walking. Patients' compliance to supplementation, renal-specific diet, and physical activity program was evaluated by phone calls twice a month. It was reconsidered by counting the returned capsules and assessing the 24-h dietary recall, 3-day food record (two weekdays and one weekend day), and international physical activity questionnaire-short form (IPAQ-SF) monthly. All patients were encouraged to adhere to intervention protocol regarding regular consumption of the capsules, following the physical activity program and renal diet at each phone call and interval visits. Participants taking less than 80% of supplementation dosage were excluded.

### Outcomes and assessments

The primary outcome of the current randomized clinical trial (RCT) was the changes in some kidney function parameters, and the secondary outcomes were changes in PAB, glycemic status, quality of life, and BP from baseline to the end of the intervention.

The socio-demographic questionnaire was filled by an interview in the first visit. Anthropometric parameters such as weight and height were assessed by validated tools at the baseline and the end of the trial. Weight was measured with light clothes and no shoes, close to 100 g, by a digital Seca scale (Seca 22089, Hamburg, Germany). Height was measured using a portable stadiometer (Seca, Hamburg, Germany), in a straight standing position and without shoes, close to 0.5 cm. Then, BMI was calculated by dividing weight in kilograms by height in meters squared. At the end of each medical appointment, BP was measured using a mercury sphygmomanometer (ALPK2, Japan). 10 mL of blood sample was obtained from each participant after 12 h of overnight fasting at the commencement of the study and at the end of intervention. Samples were collected into the tubes containing ethylenediamine tetraacetic acid (EDTA) for Hemoglobin A1C (HbA1C) analysis and tubes without anticoagulant (for centrifugation and obtaining the serum). Separated serums were used to measure insulin (enzyme-linked immunosorbent assay (ELISA), Monobind), fasting blood sugar (FBS) (enzymatic-colorimetric, Mancompany), and creatinine (Jaffe, Parsazmun). The other aliquoted serum samples were frozen at − 20 °C for the PAB assay analysis (ELISA, Merck KGaA). Hemoglobin A1C (corrected-enzymatic, Biorexfars) was determined from whole blood samples. The 24-h urine samples were collected to measure volume and the protein content (Photometric, Parsazmun) at the baseline and the end of the study. All biochemical measurements except PAB were performed immediately after sampling. Pro-oxidant–antioxidant balance assay was done as described by Faraji-Rad et al.^17^. Insulin sensitivity and insulin resistance were calculated by quantitative insulin sensitivity check index (QUICKI) and homeostasis model of assessment-insulin resistance (HOMA-IR), respectively^18,19^:

$$ {\text{QUICKI}} = {1}/\left( {{\text{log}}\,\left( {{\text{fasting}}\;{\text{serum}}\;{\text{insulin}}\,\mu {\text{IU}}/{\text{mL}}} \right) + {\text{log}}\;\left( {{\text{fasting}}\;{\text{glucose}}\,{\text{mg}}/{\text{dL}}} \right)} \right) $$

$$ {\text{HOMA}} - {\text{IR}} = {\text{fasting}}\;{\text{serum}}\,{\text{insulin}}\;\left( {\mu {\text{IU}}/{\text{mL}}} \right) \times {\text{fasting}}\;{\text{glucose}}\,\left( {{\text{mmol}}/{\text{L}}} \right)/{22}.{5} $$

#### Health-related quality of life

Health-related quality of life was evaluated with the Kidney Disease and Quality of Life Short-Form (KDQOL-${\mathrm{SF}}^{\mathrm{TM}}$, v. 1.3) questionnaire at the baseline and the end of the study. Researchers blinded to the treatment assignment verbally administered the questionnaire by an interview and recorded the responses since high illiteracy rates in the patients. The Persian version of the KDQOL-${\mathrm{SF}}^{\mathrm{TM}}$, v. 1.3 questionnaire has been translated and validated by Pakpour et al.^20^. This questionnaire consists of 36 questions about the general health status from both mental and physical dimensions and 43 kidney disease-specific questions. Responses were transported to an excel spreadsheet provided by the website (www.rand.org/health/surveys_tools/kdqol.html). This worksheet recodifies data from any items of the questionnaire, resulting in a standardized scale ranging from 0 (worst quality of life) to 100 (best quality of life). Dimensions have been scored separately, and there is no single value as a result of overall assessment of the KDQOL-${\mathrm{SF}}^{\mathrm{TM}}$, v. 1.3 questionnaire.

### Statistical analysis

Statistical analyses were conducted using IBM SPSS Statistics software, version 25 (SPSS Inc., and Chicago, IL, USA). The data entry was checked double times by the authors. Missing data were completed using a multiple imputation approach by chained equations with 20 iterations. The Shapiro–Wilk test was used to examine the normal distribution of variables. Results were presented as frequency (percentage) for qualitative variables, mean ± SD for normally distributed continuous data, or median (interquartile range 25–75 percentile) for values with skewed distribution. For comparing the baseline characteristics between the two groups, independent samples t-test was used for normally distributed continuous variables, and Chi-square test or Fisher's exact test for qualitative variables, and Mann–Whitney U test for non-normally distributed variables were used accordingly. Within-group changes were assessed by Paired samples t-test or Wilcoxon signed-rank test, as appropriate. Final differences between the two groups were identified using analysis of covariance (ANCOVA) or rank ANCOVA (according to normality), adjusting for age, present of diabetes, and baseline values as covariates. A *P*-value under 0.05 was considered the threshold of significance. All analyses were carried out using both original and imputed data, and the findings were compared. As the obtained results were similar, the findings related to the original analysis were reported.

## Results

**Figure 1:**
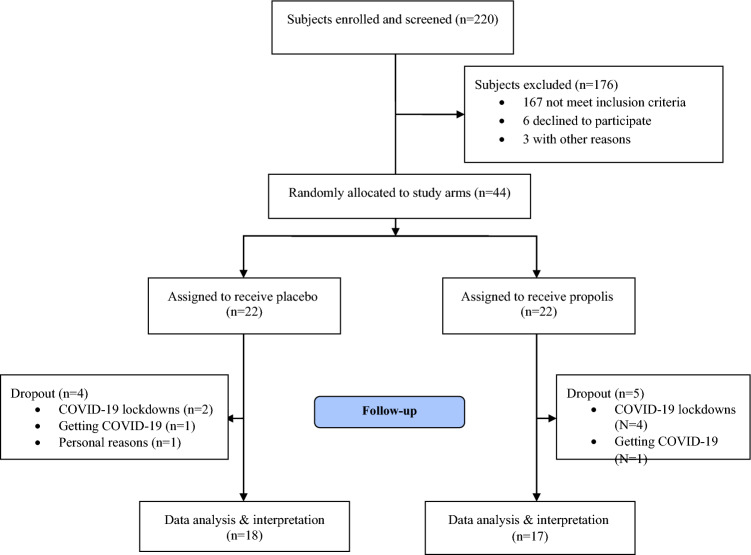
Screening, randomization, treatment, follow-up.

Of 44 patients who were included, 35 (17 in the propolis group and 18 in the placebo group) completed the study. As the present study was conducted at the peak of the coronavirus disease 2019 (COVID-19) pandemic in IRAN, four patients in the propolis group and two patients in the placebo group withdrew from the study due to COVID-19 lockdowns, and in both groups, one patient did not continue the study because of getting COVID-19; One of the patients in the placebo group also discontinued for the personal reasons. The trial flowchart was shown in Fig. 1. No serious adverse events related to participation occurred during the study. Only mild constipation in the propolis group (n = 2) and dyspepsia in the placebo group (n = 1) were reported.

The demographic and baseline characteristics of the patients were shown in Table 1. There were no statistically significant differences between the two groups in terms of age, gender, diabetes and HTN distribution, intake of BP, glucose, and lipid lowering medications; and anthropometric indices including weight, height, and BMI prior to the intervention. Furthermore, no significant differences were detected between the propolis and placebo groups at the commencement of the study regarding KDQOL-${\mathrm{SF}}^{\mathrm{TM}}$, v. 1.3 questionnaire dimensions, systolic/diastolic BP, and all biochemical data except HbA1C (Tables 2 and 3).

**Table 1:** Demographic and general characteristic of the patients at baseline.

Variables	Propolis group (*n* = 17)	Placebo group (*n* = 18)	*P*-value
Quantitative variables			
Age, years	58.06 ± 13.29	60.50 ± 11.04	0.557
Body height, cm	165.32 ± 9.29	164.94 ± 7.63	0.896
Body Weight, kg	81.09 ± 14.73	78.25 ± 14.97	0.576
Body mass index, kg/m^2^	29.66 ± 4.65	28.53 ± 3.76	0.434
Qualitative variables (*n* (%))			
Gender, male	13 (76.50)	11 (61.10)	0.328
Co-morbidities (*n* (%))			
Hypertension − / +	16 (94.10)	16 (88.90)	1.000*
Diabetes − / +	5 (29.40)	6 (33.30)	0.803
Medications (*n* (%))			
Insulin−/+	1(5.90)	2 (11.10)	1.000*
Biguanides−/+	2 (11.80)	3 (16.70)	1.000*
Sulfonylureas−/+	2 (11.80)	2 (11.10)	1.000*
SGLT2 inhibitors−/+	2 (11.80)	2 (11.10)	1.000*
Thiazolidinediones−/+	2 (11.80)	0	0.229*
Alpha glucosidase inhibitors−/+	0	1 (5.60)	1.000*
Dipeptiyl peptidase IV inhibitors−/+	0	2 (11.10)	0.486*
Meglitinides derivatives−/+	1(5.90)	1 (5.60)	1.000*
ACE inhibitors−/+	3 (17.60)	2 (11.10)	0.658*
ARBs−/+	15 (88.20)	13 (72.20)	0.402*
Beta blockers−/+	3 (17.60)	5 (27.80)	0.691*
Calcium channel blockers−/+	5 (29.40)	2 (11.10)	0.228*
Diuretics−/+	7 (41.20)	12 (66.70)	0.130
Vasodilators−/+	3 (17.60)	1 (5.60)	0.338*
Alpha blockers−/+	1(5.90)	2 (11.10)	1.000*
Combined alpha and beta blockers−/+	2 (11.80)	1 (5.60)	0.603*
Fenofibrate−/+	1(5.90)	1 (5.60)	1.000*
Statins−/+	6 (35.30)	7 (38.90)	0.826

Data are presented as mean ± standard deviation or frequencies. *P* values were obtained from the independent-sample t test for quantitative variables and Chi-square test or Fisher's exact test for qualitative variables. SGLT2 inhibitors, sodium-glucose cotransporter-2 inhibitors; ACE inhibitors, angiotensin-converting enzyme inhibitors; ARBs, angiotensin II receptor blockers.

*Fisher's exact test.

**Table 2:** Biomarkers changes after the 3-month intervention.

Outcome variables	Propolis group			Placebo group			*P*^*3*^	Within-group change Mean (SE)		*P*^*4*^	*Partial Eta Squared*
	Before	After	*P*^*1*^	Before	After	*P*^*2*^		Propolis group	Placebo group		
Systolic blood pressure, (mmHg)	138.79 ± 19.57^5^	134.50 ± 14.18	0.394^#^	132.33 ± 11.93	134.00 ± 16.28	0.565^#^	0.300*	− 2.47 (3.46)	− 0.03 (3.34)	0.621	0.010
Diastolic blood pressure, (mmHg)	92.50 [80.00–110.00]^6^	90.00 [80.00–90.00]	0.137	90.00 [80.00–105.00]	85.00 [80.00–100.00]	0.603	0.737	− 5.83 (2.53)	− 1.55 (2.45)	0.237	0.058
Serum creatinine, (mg/dl)	1.57 ± 0.36	1.61 ± 0.45	0.523^#^	1.62 ± 0.27	1.55 ± 0.30	0.240^#^	0.696*	0.05 (0.06)	− 0.07 (0.06)	0.247^a^	0.044^a^
Urine volume, (mL/24 h)	1795.88 ± 754.59	1972.35 ± 790.17	0.132^#^	2083.33 ± 693.88	1983.33 ± 536.05	0.480^#^	0.249*	128.50 (121.46)	− 54.70 (117.92)	0.296	0.036
Proteinuria, (mg/24 h)	228.00 [81.00–1026.05]	295.00 [111.24–812.00]	0.407	135.00 [70.00–300.50]	150.00 [126.90–253.50]	0.381	0.235	− 67.19 (45.17)	− 196.57 (45.17)	0.249^a^	0.045^a^
Fasting blood sugar, (mg/dl)	90.50 [81.00–96.75]	87.50 [82.00–107.25]	0.836	92.00 [86.00–120.25]	90.50 [85.50–102.00]	0.365	0.397	− 13.59 (5.05)	− 10.31 (4.76)	0.846^a^	0.001^a^
Hemoglobin A1C, (%)	5.20 [4.90–6.02]	5.80 [5.05–6.00]	0.101	6.30 [5.40–7.55]	5.90 [5.55–6.90]	0.423	0.019	− 0.31 (0.28)	0.17 (0.27)	0.371^a^	0.029^a^
Insulin, (µIU/mL)	9.70 [5.70–17.30]	9.80 [5.52–14.50]	0.955	11.40 [8.06–16.55]	12.82 [7.87–20.60]	0.981	0.584	− 1.42 (1.39)	0.19 (1.30)	0.406	0.026
HOMAIR	2.00 [1.15–3.84]	2.23 [1.21–3.74]	0.820	3.29 [1.71–5.50]	3.17 [2.00–4.58]	0.831	0.336	− 1.05 (0.43)	− 0.48 (0.40)	0.344	0.033
QUICKI	0.34 ± 0.04	0.34 ± 0.03	0.844^#^	0.33 ± 0.03	0.33 ± 0.02	0.980^#^	0.396*	0.005 (0.006)	− 0.003 (0.006)	0.387	0.028
PAB	125.83 ± 25.41	135.83 ± 35.30	0.207^#^	130.08 ± 30.95	122.09 ± 32.87	0.410^#^	0.687*	8.93 (8.67)	− 7.05 (8.10)	0.192	0.067

*SE* standard error, *HOMA-IR* homeostasis model of assessment-insulin resistance, *QUICKI* quantitative insulin sensitivity check index, *PAB* pro-oxidant–antioxidant balance.

*Based on independent samples t test.

^#^Based on paired-sample t test.

^1^Based on Wilcoxon rank-sum test (unless otherwise indicated) for within propolis group differences.

^2^Based on Wilcoxon rank-sum test (unless otherwise indicated) for within placebo group differences.

^3^Based on Mann–Whitney U test (unless otherwise indicated) for differences at baseline between propolis and placebo groups.

^4^Based on ANCOVA test (unless otherwise indicated) adjusted for age, present of diabetes, and baseline values as the covariates.

^5^Mean ± standard deviation (quantitative variables with normal distribution).

^6^Median [interquartile range] (quantitative variables with non-normal distribution).

^a^Based on rank ANCOVA.

**Table 3:** KDQOL-${\mathrm{SF}}^{\mathrm{TM}}$, v. 1.3 questionnaire dimensions after the 3-month intervention.

Outcome variables	Propolis group			Placebo group			*P*^*3*^	Within-group change Mean (SE)		*P*^*4*^	*Partial Eta Squared*
	Before	After	*P*^*1*^	Before	After	*P*^*2*^		Propolis group	Placebo group		
Symptom problem list	87.50 [80.12–94.89] ^6^	98.86 [85.80–100.00]	0.018	84.09 [75.00–90.91]	86.36 [76.13–93.18]	0.312	0.505	10.98 (2.31)	2.68 (1.94)	0.002^a^	0.323
Effects of kidney disease	85.71 [59.37–100.00]	95.83 [84.37–100.00]	0.036	92.26 [83.92–98.96]	91.67 [80.21–95.83]	0.700	0.301	8.22 (3.44)	2.20 (2.95)	0.111^a^	0.107
Burden of kidney disease	81.25 [40.62–100.00]	87.50 [40.62–100.00]	0.944	68.75 [50.00–96.87]	75.00 [59.37–100.00]	0.455	0.875	1.26 (5.81)	4.63 (4.87)	0.662	0.008
Work status	50.00 [50.00–100.00]	50.00 [50.00–87.50]	0.564	50.00 [50.00–50.00]	50.00 [50.00–50.00]	0.083	0.515	− 3.26 (5.45)	− 10.05 (4.71)	0.359	0.037
Cognitive function	66.67 [48.33–83.33]	73.33 [66.67–86.67]	0.108	73.33 [53.33–86.67]	73.33 [60.00–76.66]	0.529	0.738	11.07 (4.35)	3.16 (3.65)	0.179	0.074
Quality of social interaction	80.00 [55.00–86.67]	86.67 [61.67–98.33]	0.311	76.66 [53.33–98.33)	76.66 [61.67–86.67]	0.950	0.623	6.11 (5.67)	− 2.29 (4.89)	0.160^a^	0.084
Sleep	73.75 [44.37–94.37]	76.25 [42.50–96.25]	0.935	75.00 [30.00–83.75]	75.00 [63.75–91.25]	0.129	0.564	1.78 (5.97)	7.13 (5.00)	0.502	0.019
Social support	100.00 [75.00–100.00]	100.00 [75.00–100.00]	0.564	100.00 [91.66–100.00]	100.00 [100.00–100.00]	0.833	0.723	1.29 (6.92)	− 0.91 (5.78)	0.707^a^	0.006
Overall health	60.00 [40.00–75.00]	80.00 [65.00–80.00]	0.029	60.00 [50.00–80.00]	70.00 [50.00–70.00]	0.936	0.356*	12.60 (3.64)	1.63 (3.27)	0.038	0.168
Patient satisfaction	76.00 [66.67–100.00]	76.50 [71.58–83.33]	0.589	76.00 [69.50–83.33]	79.91 [74.04–100.00]	0.426	0.761	− 1.00 (5.06)	1.93 (4.92)	0.734^a^	0.004
Physical functioning	90.00 [65.00–100.00]	97.50 [85.00–100.00]	0.157	90.00 [82.50–100.00]	95.00 [82.50–97.50]	0.547	0.928	4.13 (3.30)	2.38 (2.77)	0.309^a^	0.043
Role physical	100.00 [0.00–100.00]	100.00 [100.00–100.00]	0.046	100.00 [87.50–100.00]	100.00 [100.00–100.00]	0.581	0.249	24.84 (9.02)	10.41 (7.53)	0.159^a^	0.081
Pain	85.00 [46.25–100.00]	78.75 [50.62–100.00]	0.765	100.00 [57.50–100.00]	80.00 [43.75–100.00]	0.168	0.438	1.00 (5.83)	− 9.53 (4.87)	0.267^a^	0.051
General health	60.00 [25.00–75.00]	80.00 [65.00–85.00]	0.014	65.00 [47.50–80.00]	60.00 [42.50–80.00]	0.962	0.351	15.60 (4.93)	1.04 (4.12)	0.035	0.172
Emotional well being	57.33 ± 34.04 ^5^	65.33 ± 25.20	0.330^#^	66.35 ± 21.40	62.11 ± 23.15	0.418^#^	0.388*	6.73 (5.60)	− 3.34 (4.68)	0.185	0.072
Role emotional	33.33 [0.00–100.00]	100.00 [50.00–100.00]	0.067	100.00 [33.33–100.00]	100.00 [16.66–100.00]	0.719	0.096	16.30 (13.03)	6.14 (10.86)	0.508^a^	0.018
Social function	75.00 [53.12–100.00]	100.00 [71.87–100.00]	0.076	100.00 [68.75–100.00]	100.00 [75.00–100.00]	0.797	0.157	11.52 (5.37)	2.90 (4.48)	0.101^a^	0.108
Energy fatigue	65.00 [36.25–88.75]	73.33 [61.25–88.75]	0.123	60.00 [47.50–85.00]	70.00 [55.00–72.50]	0.917	0.830*	11.10 (4.32)	0.89 (3.63)	0.084	0.119
SF12 Physical composite	49.30 [45.32–54.21]	53.49 [51.42–56.58]	0.286	48.21 [39.18–52.31]	53.32 [40.08–56.05]	0.152	0.310	4.56 (2.10)	2.11 (1.93)	0.409	0.036
SF12 Mental composite	46.00 [19.41–55.96]	53.08 [34.44–58.63]	0.155	49.16 [40.89–55.37]	48.04 [37.49–54.47]	0.279	0.505	5.55 (3.93)	− 1.91 (3.59)	0.195	0.087

KDQOL-${\mathrm{SF}}^{\mathrm{TM}}$, v. 1.3, kidney disease and quality of life short-form.

*Based on independent samples t test.

^#^Based on paired-sample t test.

^1^Based on Wilcoxon rank-sum test (unless otherwise indicated) for within propolis group differences.

^2^Based on Wilcoxon rank-sum test (unless otherwise indicated) for within placebo group differences.

^3^Based on Mann–Whitney U test (unless otherwise indicated) for differences at baseline between propolis and placebo groups.

^4^Based on ANCOVA test (unless otherwise indicated) adjusted for age, present of diabetes, and baseline values as the covariates.

^5^Mean ± standard deviation (quantitative variables with normal distribution).

^6^Median [interquartile range] (quantitative variables with non-normal distribution).

^a^Based on rank ANCOVA.

At the end of the intervention, systolic and diastolic BP values decreased non-significantly in both groups, while the propolis group showed higher reductions (Table 2). No significant between-group differences were observed in mean systolic and diastolic BP changes throughout the trial, even after adjusting baseline values, present of diabetes, and age (Table 2). Biochemical parameters were reported in Table 2. The results showed that the differences between the two groups in terms of mean changes of glycemic indices were not statistically significant. Furthermore, within group changes of glycemic indices were not significant in both study groups as well, with some non-statistically significant lowering effects of the propolis. The mean differences in serum creatinine, 24-h urine volume and protein, and PAB over the course of the study were not remarkable between the two groups.

The KDQOL-${\mathrm{SF}}^{\mathrm{TM}}$, v. 1.3 questionnaire dimensions were shown in Table 3. Comparing the baseline and the end-point values in the propolis group, a significant improvement was observed in the following components of the quality of life; symptoms/problems (*P* = 0.018), effects of kidney disease (*P* = 0.036), overall health (*P* = 0.029), physical performance (*P* = 0.046), and general health (*P* = 0.014). Notably, the control group showed no significant changes in all the dimensions of the KDQOL-${\mathrm{SF}}^{\mathrm{TM}}$, v. 1.3 questionnaire at the same point time. At the end of the twelfth week of intervention, the propolis group improved significantly in domains of symptoms/problems (*P* = 0.002), overall health (*P* = 0.038), and general health (*P* = 0.035) adjusting for the baseline values, present of diabetes, and age, compared to the placebo group (Table 3).

## Discussion

In the current study, CKD patients with a moderate loss of renal function due to different conditions were studied. The propolis supplementation at a dose of 250 mg daily for three months significantly improved some dimensions of HRQoL in patients with CKD (symptoms/problems, overall health, and general health). To the best of our knowledge, this is the first clinical trial investigating the effect of propolis supplementation on quality of life, PAB, renal function and glycemic parameters, and BP in CKD patients.

Oxidative stress is defined as a state of imbalance between pro-oxidants and antioxidants in favor of pro-oxidants^17^. It plays a pivotal role in the progression of CKD, directly by inducing tubular and glomerular injury or indirectly by developing HTN, inflammation, and/or endothelial dysfunction^11,21^; therefore, the effects of propolis on OS in CKD patients was evaluated in the current study. In a review by Kocot et al., it has been stated that propolis, as one of the richest sources of plant-based polyphenols, including flavonoids, can neutralize the effects of OS, which plays a detrimental role in the pathogenesis of several diseases^10^. In the study by Mujica et al., propolis administration for 90 days in a human population in Chile resulted in the increased serum levels of glutathione (GSH) and decreased thiobarbituric acid reactive substance (TBARS) levels, as a strong indicator for cardiovascular events^22^. In another study, Hesami et al. conducted a double-blind, placebo-controlled clinical trial on type 2 diabetic patients and reported that in the propolis group compared to the placebo, the level of oxidized low-density lipoprotein cholesterol (Ox-LDL-C) reduced and catalase (CAT) activity improved significantly^23^. In addition, in the studies by Gao et al. and Zhao et al. on type 2 diabetes mellitus (T2DM) patients, administration of propolis for 18 weeks caused a significant increase in serum GSH and total polyphenols in comparison to the control; however, the ferric-reducing ability of plasma (FRAP), superoxide dismutase (SOD), glutathione peroxidase (GPx), malondialdehyde (MDA) or Ox-LDL-C levels were not affected by propolis supplementation^24,25^. As mentioned in our systematic review, the experimental studies came to the conclusion that propolis could be effective in decreasing the MDA levels in serum, urine, as well as renal and liver tissues and it may increase antioxidant parameters such as serum SOD and glutathione-S-transferase (GST), liver GPx, and renal GSH, CAT, FRAP, paraoxonase (PON1), heme-oxygenase-1(HO-1) score, and total antioxidant status (TAS) in the rat models with CKD or acute kidney injury (AKI) caused by diabetes, HTN, ischemic-reperfusion, or partial nephrectomy^11^. Moreover, experimental studies showed that propolis had potential effects on reducing urinary levels of TBARS, the levels of renal tissue total oxidant status (TOS) and oxidative stress index (OSI), as well as 8-hydroxy-2′-deoxyguanosine (8-OHdG) generation, as a notable biomarker of DNA damage in the kidney tissues^11^. Nevertheless, propolis supplementation had no significant effect on serum GPx and liver CAT in these animal studies. The current study showed that propolis had no considerable impact on PAB in patients with CKD. As antioxidant properties of propolis strongly depend on its dosage and polyphenol contents^26^, various propolis obtained from different geographical areas with diverse plant species may be responsible for these controversies in outcomes across studies. Moreover, the components of the antioxidant system, such as antioxidant enzymes as well as MDA as a reactive compound and strong marker for oxidative stress, were not investigated separately in this study, and some of these components may have changed in line with the improvement of the antioxidant status, although not so much that it caused the change of PAB. Differences in the health and nutrition status of the participants at the baseline could be another reason for this inconsistency.

Chronic hyperglycemia is the major cause of micro-and macro-vascular complications associated with diabetes mellitus and the leading reason for CKD^11,27^. Hyperglycemia leads to renal lesions through multiple mechanisms, including stimulating the reactive oxygen species (ROS)-mediated pathways such as nuclear factor kappa B (NF-κB) activation, angiotensin II synthesis, protein kinase C (PKC), hexosamine pathway flux, polyol pathway flux, and advanced glycation end products (AGEs) formation^11^. As good glycemic control can prevent the initiation and progression of CKD, the effects of propolis on glycemic indices were addressed in this study. Obtained results showed that propolis had no significant effect on FBS, serum insulin, HbA1C, insulin sensitivity, and insulin resistance. In line with our results, two clinical trials reported that administration of propolis to patients with CKD (500 mg/day for one year)^14^ or nonalcoholic fatty liver disease (NAFLD) (500 mg/day for four months)^28^ had not any significant effects on glucose metabolism. In addition, the studies by Mujica et al., Gao et al., Zhao et al., and Fukuda et al. showed no significant effects of propolis administration on FBS, insulin, HbA1C, and HOMA-IR in patients with T2DM or cardio-metabolic abnormalities^16,22,24,25^. Conversely, Koo et al. reported that propolis supplementation (600 mg/day for four weeks) in healthy smokers reduced serum levels of FBS^29^. Moreover, Afsharpour et al. demonstrated that propolis supplementation reduced FBS, 2-h postprandial glucose (2hpp Glc), insulin, insulin resistance, and HbA1C at a daily dose of 1500 mg after eight weeks of intervention in T2DM patients^30^. Also, the study of Zakerkish et al. revealed that 1000 mg/day of propolis for 90 days could significantly decrease HbA1C, insulin, and 2hpp Glc levels and increase insulin sensitivity in T2DM patients, while it did not affect FBS^31^. A recent meta-analysis evaluated the effectiveness of propolis supplementation on markers of glycemic status in patients with T2DM^32^. It illustrated that propolis supplementation could significantly lower FBS and HbA1C levels; However, it had no effects on serum insulin concentrations and insulin resistance. Another meta-analysis conducted on different health populations revealed that propolis consumption decreased FBS, HbA1C, and insulin levels while not improving HOMA-IR^33^. Patients with CKD usually have T2DM as a concomitant and/or underlying disease, but few participants were diabetic in this trial. It seems that, existing inconsistency may be due to differences in the study population. The possible properties of propolis to exert glucose-lowering effects is suggested to be due to the increased insulin production and/or sensitivity, inhibitory effects on α-glycosidase and intestinal sucrose, increased glucose uptake as well as translocation of insulin-sensitive glucose transporter (GLUT) 4 via inducing phosphorylation of both phosphatidylinositol 3-kinase (PI3K) and 5’-adenosine monophosphate-activated protein kinase (AMPK), down-regulation of gluconeogenic genes in the liver, and increased glucose utilization and glycolysis in the hepatocellular cells^11^.

This study also showed that propolis had no significant effects on BP as one of the leading causes of CKD. In accordance with our findings, Silveira et al. reported that propolis supplements at a daily dose of 500 mg for one year had no effect on BP in CKD patients^14^. Contrary to our findings, experimental studies have shown the antihypertensive effects of propolis. Angiotensin-converting enzyme inhibitor-like effect, diuretic effects, activation of nitric oxide pathway, acetylcholine-induced vasodilation, and the antioxidant and anti-inflammatory properties of propolis are among the suggested mechanisms^12,26,34–40^. The vasorelaxant effects of propolis also occur as a result of inhibitory action on calcium movements through smooth muscle cells membrane^40^. Different results obtained may be due to the fact that the hypertensive patients of the current study were taking antihypertensive medications, as seen in the study of Silveira et al.^14^

Only few studies have been carried out using CKD models. Silveira et al. reported that daily consumption of 500 mg propolis extract for one year in patients with CKD significantly reduced proteinuria but not plasma creatinine^14^. Likewise, in another study, 1000 mg/day of propolis administration for 90 days in T2DM patients had no effects on serum creatinine^31^. Teles et al. have reported renal protective properties of the alcoholic extract of red propolis (150 mg/kg/day for two months) in hypertensive rats with CKD and proteinuria (5/6 renal ablated models), manifested by reducing proteinuria, serum concentrations of creatinine, glomerulosclerosis, and renal macrophage infiltration^12^. Moreover, it has been demonstrated that oral administration of chrysin (10 mg/kg/day, for ten weeks), one of the flavonoid compounds found in propolis, can attenuate proteinuria, glomerular injury, and podocyte apoptotic loss due to exposure to high levels of glucose in diabetic rats^13^. In the present clinical trial, we did not find a statistically significant effect of propolis on serum levels of creatinine, proteinuria, and urine volume, although 3-month intervention period may be too short to assess CKD progression through the measurement of serum creatinine concentrations and proteinuria, which has well-known limitations.

The present study showed that propolis supplementation at a dose of 250 mg daily for three months improved some dimensions of HRQoL in patients with CKD (symptoms/problems, overall health, and general health). Davoodi et al. also found that propolis administration (250 mg/day for three months) in patients diagnosed with breast cancer receiving chemotherapy improved the quality of life, particularly with regards to global quality of life as well as emotional functioning and financial difficulties compared to the placebo group^41^. Nonetheless, in the study by Matsumoto et al., after 24 weeks of intervention with propolis at a daily dose of 508.5 mg in women with rheumatoid arthritis, no significant difference was observed in the quality of life^42^. As there are no consistent results in this regard, without any clear underlying mechanisms, more clinical trials are needed to draw robust conclusions about propolis efficacy in HRQoL for patients with CKD.

There were some limitations in this research. The chemical profile of the propolis extract was determined by the manufacturer laboratory in Isfahan, Iran, including general information, and the effective components and their exact values within each capsule were not identified. Additionally, serum levels of propolis bioactive compounds were not measured for assessing the patients’ compliance to propolis supplementation; however, compliance rate was measured by counting the returned capsules by the participants during the pre-arranged appointments. Notably, one of the challenges we faced was that the study was conducted at the peak of the COVID-19 pandemic in IRAN, so some participants may had asymptomatic COVID-19 infection, that affected the results. The relatively low dose of porpolis—due to safety considerations for CKD patients—and the short duration of intervention, which may be the reasons for the lack of significant changes in serum creatinine and proteinuria, are other limitations of this study. All patients had stages 2 and 3 of CKD, so the generalizability of these results in patients with more advanced stages of CKD needs to be confirmed in future clinical trials. However, attempts were made to overcome the impact of limitations by stratified block randomization with a block size of two (based on age and present of diabetes), adjusting the results for confounding factors, and involvement of patients with CKD of different causes.

In conclusion, treatment with Iranian poplar type propolis may improve HRQoL in patients with CKD of any causes and moderate renal dysfunction. These results indicated the therapeutic effects of Iranian poplar type propolis in this group of patients, and opened a window for further researches on this compound with larger sample size, longer duration, and higher dosage, considering propolis as an adjuvant therapy in CKD patients.

## Supplementary Information

Supplementary Information.

## Data Availability

Data is available upon request submitted to the corresponding author.

## Abbreviations

CKD: Chronic kidney disease
HTN: Hypertension
eGFR: Estimated glomerular filtration rate
ESRD: End-stage renal disease
DALYs: Disability-adjusted life-years
CVD: Cardiovascular disease
HRQoL: Health-related quality of life
OS: Oxidative stress
PAB: Prooxidant-antioxidant balance
BP: Blood pressure
BMI: Body mass index
SD: Standard deviation
DNP: 2,4- Dinitrophenylhydrazine
IPAQ-SF: International physical activity questionnaire-short form
RCT: Randomized clinical trial
EDTA: Ethylenediamine tetraacetic acid
HbA1C: Hemoglobin A1C
ELISA: Enzyme-linked immunosorbent assay
FBS: Fasting blood sugar
QUICKI: Quantitative insulin sensitivity check index
HOMA-IR: Homeostatic model assessment for insulin resistance
KDQOL-SF: Kidney Disease and Quality of Life-Short Form
ANCOVA: Analysis of covariance
COVID-19: Coronavirus disease 2019
GSH: Glutathione
TBARS: Thiobarbituric acid reactive substance
Ox-LDL-C: Oxidized low-density lipoprotein cholesterol
CAT: Catalase
T2DM: Type 2 diabetes mellitus
FRAP: Ferric-reducing ability of plasma
SOD: Superoxide dismutase
GPx: Glutathione peroxidase
MDA: Malondialdehyde
GST: Glutathione-S-transferase
PON1: Paraoxonase
HO-1: Heme-oxygenase-1
TAS: Total antioxidant status
AKI: Acute kidney injury
TOS: Total oxidant status
OSI: Oxidative stress index
8-OHdG: 8-Hydroxy-2′-deoxyguanosine
ROS: Reactive oxygen species
NF-κB: Nuclear factor kappa
PKC: Protein kinase C
AGEs: Advanced glycation end products
NAFLD: Nonalcoholic fatty liver disease
2hpp Glc: 2-Hour postprandial glucose
GLUT: Insulin-sensitive glucose transporter
PI3K: Phosphatidylinositol 3-kinase
AMPK: 5’-Adenosine monophosphate-activated protein kinase
